# Supplementary material for: A fault-intrusion-tolerant system and deadline-aware algorithm for scheduling scientific workflow in the cloud
Source: PeerJ Comput Sci. 2021 Nov 2;7:e747. doi: 10.7717/peerj-cs.747 (PMC8576554; doi:10.7717/peerj-cs.747)
Supplement: Supplemental Information 6 [file peerj-cs-07-747-s006.zip › Myproject1.1/docs/org/cloudbus/cloudsim/Packet.html]

Packet


JavaScript is disabled on your browser.


- Overview
- Package
- Class
- Use
- Tree
- Deprecated
- Index
- Help

- Prev Class
- Next Class

- Frames
- No Frames

- All Classes

- Summary:
- Nested |
- Field |
- Constr |
- Method

- Detail:
- Field |
- Constr |
- Method


org.cloudbus.cloudsim

## Interface Packet

- All Known Implementing Classes:
  :   InfoPacket

  ---

    

  ```
  public interface Packet
  ```

  This class contains the structure for a network packet.

  Since:
  :   CloudSim Toolkit 1.0

- - ### Method Summary

    Methods

    | Modifier and Type | Method and Description |
    | --- | --- |
    | `int` | `getDestId()` Returns the destination id of this packet. |
    | `int` | `getId()` Returns the ID of this packet |
    | `int` | `getLast()` Gets an entity ID from the last hop that this packet has traversed. |
    | `int` | `getNetServiceType()` Gets the network service type of this packet |
    | `long` | `getSize()` Returns the size of this packet |
    | `int` | `getSrcId()` Returns the ID of the source of this packet. |
    | `int` | `getTag()` Gets this packet tag |
    | `void` | `setLast(int last)` Sets an entity ID from the last hop that this packet has traversed. |
    | `void` | `setNetServiceType(int serviceType)` Sets the network service type of this packet. |
    | `boolean` | `setSize(long size)` Sets the size of this packet |
    | `java.lang.String` | `toString()` Returns a string describing this packet in detail. |

- - ### Method Detail


    - #### toString

      ```
      java.lang.String toString()
      ```

      Returns a string describing this packet in detail.

      **Overrides:**
      :   `toString` in class `java.lang.Object`

      Returns:
      :   description of this packet


    - #### getSize

      ```
      long getSize()
      ```

      Returns the size of this packet

      Returns:
      :   size of the packet


    - #### setSize

      ```
      boolean setSize(long size)
      ```

      Sets the size of this packet

      Parameters:
      :   `size` - size of the packet

      Returns:
      :   true if it is successful, false otherwise


    - #### getDestId

      ```
      int getDestId()
      ```

      Returns the destination id of this packet.

      Returns:
      :   destination id


    - #### getId

      ```
      int getId()
      ```

      Returns the ID of this packet

      Returns:
      :   packet ID


    - #### getSrcId

      ```
      int getSrcId()
      ```

      Returns the ID of the source of this packet.

      Returns:
      :   source id


    - #### getNetServiceType

      ```
      int getNetServiceType()
      ```

      Gets the network service type of this packet

      Returns:
      :   the network service type


    - #### setNetServiceType

      ```
      void setNetServiceType(int serviceType)
      ```

      Sets the network service type of this packet.

      By default, the service type is 0 (zero). It is depends on the packet scheduler to determine
      the priority of this service level.

      Parameters:
      :   `serviceType` - this packet's service type


    - #### getLast

      ```
      int getLast()
      ```

      Gets an entity ID from the last hop that this packet has traversed.

      Returns:
      :   an entity ID


    - #### setLast

      ```
      void setLast(int last)
      ```

      Sets an entity ID from the last hop that this packet has traversed.

      Parameters:
      :   `last` - an entity ID from the last hop


    - #### getTag

      ```
      int getTag()
      ```

      Gets this packet tag

      Returns:
      :   this packet tag


- Overview
- Package
- Class
- Use
- Tree
- Deprecated
- Index
- Help

- Prev Class
- Next Class

- Frames
- No Frames

- All Classes

- Summary:
- Nested |
- Field |
- Constr |
- Method

- Detail:
- Field |
- Constr |
- Method
